# Concurrent Validity and Reliability of Devices to Measure Jump Height in Men’s Handball Players

**DOI:** 10.3390/s22239070

**Published:** 2022-11-23

**Authors:** Alejandro Soler-López, Antonio García-de-Alcaraz, Adrián Moreno-Villanueva, José Pino-Ortega

**Affiliations:** 1 Faculty of Sports Sciences, University of Murcia, 30720 San Javier, Spain; 2 Faculty of Educational Sciences, University of Almería, 04120 Almería, Spain; 3 SPORT Research Group (CTS-1024), CERNEP Research Center, University of Almería, 04120 Almería, Spain; 4 BIOVETMED & SPORTSCI Research Group, Department of Physical Activity and Sport, Faculty of Sport Sciences, University of Murcia, 30720 San Javier, Spain

**Keywords:** team sports, stretch-shortening cycle, inertial devices, wearable, microtechnology, testing, reproducibility

## Abstract

Although there is a wide range of validated devices to measure vertical jump height, the degree of interchangeability among them is currently unknown. Aims: The purpose of this study was to examine the concurrent validity and reliability of multiple devices to measure jump height in men’s handball players. Methods: Sixteen players (age = 24.0 ± 3.7 years old) performed three types of jumps (n= 144—squat jump (SJ), countermovement jump (CMJ) and Abalakov jump (ABK)) on a contact platform (CHRONOJUMP^®^) while simultaneously being measured with two inertial devices (WIMU^®^ and VERT^®^) and recorded with a high-speed camera. Vertical jump height was analyzed according to each type of jump. Results: The t-test showed statistically significant differences (*p* = 0.001) between the contact platform (reference standard) and the rest of the tools that tended to overestimate jump height in all jumps. SJ and CMJ proved to be the jump tests with the most stable reliability values in all devices (ICC: 0.92–0.98), except in the comparison with VERT^®^. Conclusions: Although all the analyzed devices proved to be valid and reliable in previous studies, they are not interchangeable. Therefore, it is suggested to always use the same type of device to evaluate vertical height jump.

## 1. Introduction

Handball is a high-intensity body contact sport that demands a high level of aerobic and anaerobic fitness [1], also requiring speed, power, strength, agility, and endurance during the match [2]. These attributes emerge in game actions such as jumps. Jumps constitute one of the most determining physical–technical elements in the performance of a handball match, both in offensive and defensive actions [3,4,5]. In professional handball, the jump throw is the most common throw, representing over 70% of all throws in a game situation [6]. In addition to throwing velocity and accuracy, vertical jump height is potentially an important performance factor in a jump throw. A greater jump height affords any player, regardless of playing position, more throwing opportunities as a function of either position or time spent in the air [7]. Therefore, a very common training goal is to improve jumping ability [1]. Plyometric exercises with quick and powerful multi-joint movements (jumping, hopping, skipping, etc.) are performed for that purpose [8,9,10]. Vertical jump tests are used by trainers to measure the enhancements in jump skill [11,12,13,14]. Thus, jump height is one of the most commonly used variables to evaluate athletes’ performance because it is an indicator of lower limb muscle power [15] and neuromuscular fatigue [16,17], with a strong negative correlation (*r* > −0.90) with indices of exercise exhaustion and stress such as blood lactate [17], ammonia [17] and salivary free cortisol concentrations [18].

Several approaches have been developed to measure jump height. High-speed cameras have high accuracy, but are extremely time-consuming [19]. Force platforms have been considered as the gold standard [20,21,22,23]. Then, contact platforms measure the vertical jump through the time the athlete is in the air [24,25], being a highly valid and reliable method too [20,25]. However, these two systems cannot be used to calculate jump height during competition or training, but only under laboratory conditions (controlled and standardized environment), and nowadays, accelerometers are used to calculate flight time, and can collect jump height data in real time from training or competition. [11,20,22,26,27,28]. Although there is an obvious difficulty in calculating the flight time, most devices use this variable. Due to the wide range of options, it is important to select and use them properly according to precision, cost, reliability or duration [29], and also in terms of quick data readiness.

Although contact platforms such as Chronojump (Boscosystem^®^, Barcelona, Spain) and high-speed cameras provide a reliable and accurate assessment with higher intraclass correlation coefficients (0.999 and 0.997, respectively) [30,31], these devices have many limitations for measuring in real conditions (e.g., jumping during training or a match) and providing information immediately (to prevent training overreaching, help in coaching decisions, etc.). In this respect, the accelerometer is a new wearable technology that attempts to solve this limitation. Thus, the VERT^®^ (Mayfonk Athletic, Florida, USA) is a wearable that records jump count and height [32,33], while another device, the WIMU^®^ (RealTrack Systems, Almería, Spain), has been used in sports in which horizontal movements predominate, also with high intraclass correlation coefficients (0.850 and 0.970, respectively) [21,33]. Therefore, although previous research studies have evaluated jump height with these different devices, there is a lack of knowledge in terms of the evaluation of the concurrent validity and reliability among these devices. Although there is a wide range of validated devices to measure vertical jump height, the degree of interchangeability among them is currently unknown. Therefore, the aim of this study was to examine the concurrent validity and reliability of multiple devices to measure jump height in men’s handball players by determining the degree of interchangeability between the different measurement devices commonly used by coaches. The results of this research could be applied to individual or team sports in which the measurement of a vertical jump is a relevant parameter to be taken into account.

## 2. Material and Methods

### 2.1. Participants

A professional men’s handball team comprising 16 players (age = 24.0 ± 3.7 years old; height = 1.83 ± 0.08 cm; and body mass = 81 ± 10 kg) that participated in the Spanish silver league (second league) took part in the study. A total of 143 jumps were recorded (n = 47 squat jumps (SJ), 48 countermovement jumps (CMJ), and 48 Abalakov jumps (ABK)). There was one less SJ due to a loss in the recording process. The minimum sample size was 44.2 jumps per test considering a factor error of 5% and a confidence interval of 95%. Thus, the jumps recorded were enough for the established goal.

The players trained regularly (at least three times per week) and played a weekly match. The exclusion criteria were: (a) potential medical problems or a history of ankle, knee, or back pathology in the three months before the study, (b) medical or orthopedic problems that compromised their participation or performance, and (c) any lower extremity reconstructive surgery in the previous two years or unresolved musculoskeletal disorders.

All the players were informed about the risks and benefits, and all of them signed the appropriate informed consent document before the test. The study protocol complied with the Declaration of Helsinki for Humans and was approved by University of Murcia Ethics Committee.

### 2.2. Testing Procedure

**Figure 1:**
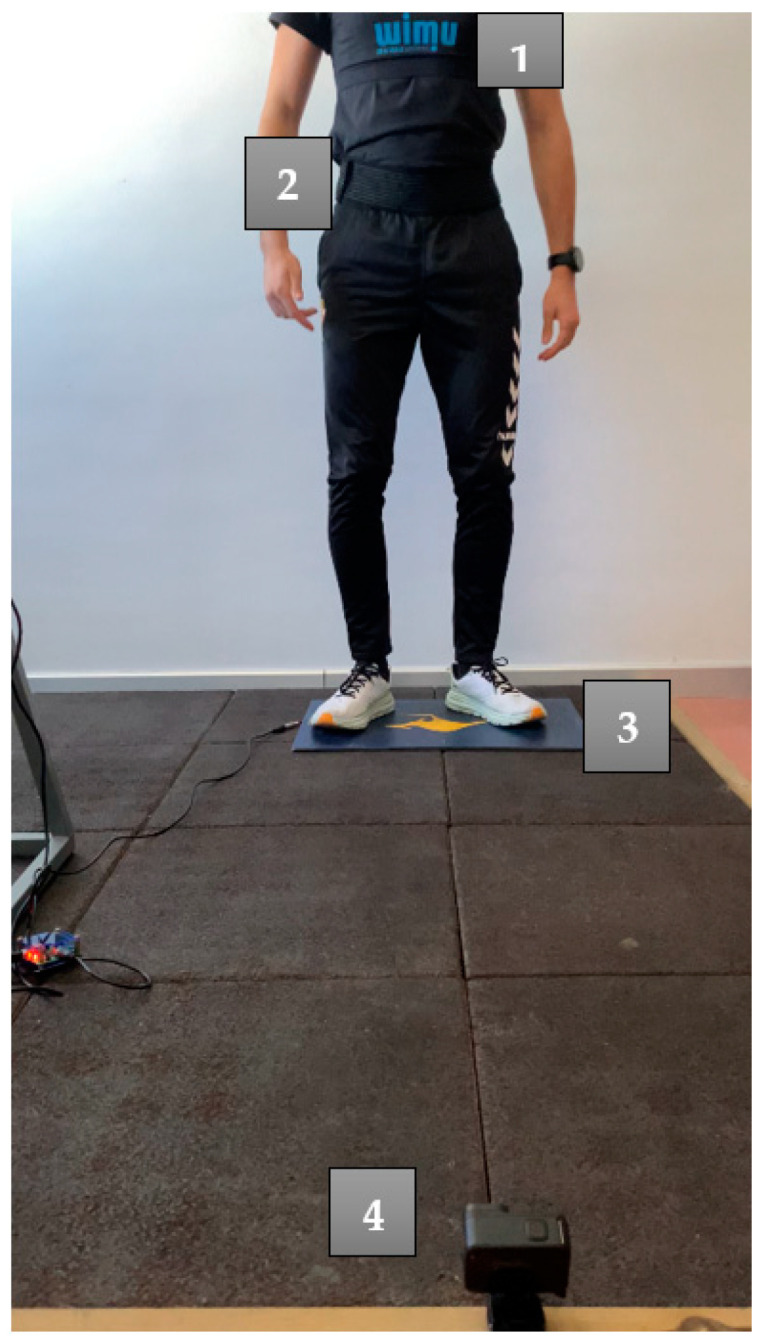
Study setup and locations of the tested devices. (**1**) WIMU PRO^TM^ (**2**) VERT^®^ (**3**) Chronojump Boscosystem^®^ (**4**) high-speed camera: A GoPro^®^ HERO9 Black HD.

The participants completed a standard 10 min warm-up composed of jogging, lower-body dynamic stretches, and vertical jumps [34]. Then, each participant performed three SJs followed by three CMJs and ABKs on a contact platform (Chronojump Boscosystem^®^, Barcelona, Spain). All the jumps were video-recorded with a high-speed camera set at 1.5 m from the platform and oriented perpendicularly to the sagittal plane of the player. Each player also wore two sensors: (a) a WIMU PRO^TM^ (Realtrack Systems, Almería, Spain) placed on the back (inter-scapula line), and (b) a VERT^®^ (Fort Lauderdale, FL, USA) placed slightly under and lateral to the participant’s umbilicus [33]. The devices were positioned as displayed in Figure 1. All participants had several years’ experience (>1 year) in jump testing, and were instructed in performing the different type of jumps (SJ, CMJ and ABK) by the same examiner. There were three seconds of rest between each jump, and five seconds between types of jumps. A longer rest was not necessary because the goal was to measure the player’s jump height at the same time for the different devices. Finally, the test was performed three days after the last game, at the same time, and with the same sport shoes and clothes.

### 2.3. Instruments

CHRONOJUMP^®^: a contact platform system composed of an A3-size (297 × 420 mm) mat with two isolated pressure-dependent electrical switches in an open-circuit configuration that closed when the athlete stepped on the mat. A PC-based (Chronojump) microcontroller was connected to the mat and computed flight time with 1-ms temporal resolution (1000 Hz). The path of the center of gravity during flight (jump height *h*) was computed by means of measured flight time (*t*) with the standardized equation *h* = *t*^2^·*g*/8. The sensitivity threshold of the platform was set to 50 ms [18,35].

WIMU^®^: Several microelectromechanical sensors (four accelerometers 2x ± 16, ± 32 and ± 400 g; 3x gyroscope at 2000°/s; and 1x magnetometer) were set at 100 Hz, as this is the minimum sampling frequency recommended for recording in sports [36]. In addition, this system can calculate the jump height using three different procedures based on acceleration, flight time, or speed.

VERT^®^: the Vert Classic (Model #JEM, Mayfonk Athletic, Fort Lauderdale, FL, USA) has a very high-precision 3-axis: gyroscope, accelerometer and magnetometer. The VERT^®^ algorithm measures take-off velocity and landing impact with over 53 simultaneous calculations to measure the vertical displacement of the center of mass. The device was connected via Bluetooth 4.0 to an Apple iPad mini 2 with the Vert Coach application (Version 2.0.6, Mayfonk Athletic, Fort Lauderdale, FL, USA). Range: 100–150 feet line-of-sight [33,35].

HIGH-SPEED CAMERA: A GoPro^®^ HERO9 Black HD at 240 frames per second was used. To measure the flight time, two independent observers trained in video analysis selected the first frame where both feet left the floor completely, and the first frame where at least one foot touched the floor again. The software’s “Timer” tool was used to obtain the final time.

### 2.4. Jump Performance

The SJ was performed from a starting position in which the athlete bent to a 90-degree knee angle; measurement was taken with the Halo Digital Goniometer (Halo Medical Devices, Sydney, Australia). The participants kept their hands on their hips, avoiding any arm swing and countermovement (pre-stretch) prior to the jump [15]. The CMJ was performed with hands on hips, starting from a static standing position and allowing the leg to make a counter movement before the jump. The same procedure was applied for the ABK jump, but allowing a full arm swing [37]. In all situations, the players were required to jump as high as possible, as well as landing at the same point on the take-off area. Moreover, the landing was performed simultaneously with feet keeping ankle dorsiflexion and avoiding knee bending for measurement alterations [15].

### 2.5. Statistical Analysis

Firstly, a descriptive analysis was performed (mean ± standard error of the mean), and the minimum detectable change (MDC) in terms of the type of jump (SJ, CMJ or ABK) for each athlete’s jump perform. The normality of the data was assessed with the Shapiro–Wilk test. Then, t-tests for paired comparisons were performed between the CHRONOJUMP^®^ and the rest of the instruments. The effect size (Cohen’s *d*) was also analyzed using the following threshold: <0.2 trivial; 0.2–0.5 small; 0.5–0.8 moderate; and >0.8 large. Moreover, the absolute agreement (interclass correlation coefficients (ICC) with a two-way mixed effect model) at a 95% confidence interval (CI) was calculated to assess the reproducibility between tools. Finally, Bland–Altman plots (showing the mean and one standard deviation above and below it) and level of agreement (R^2^) were graphically represented. In all tests, the level of significance was set at *p* < 0.05. The graphics were created using a custom Excel spreadsheet, and the statistical analyses were estimated in SPSS 25.0 (SPSS, Chicago, IL, USA).

## 3. Results

Table 1 indicates a descriptive information about the jump height in terms of the type of jump from all the actions recorded. A small standard error of mean was found (less than 1.5 cm), while the MDC was over two centimeters in almost all situations. The lowest MDC values in the SJ was observed in WIMU^®^ (acceleration and time fly) and also in the VERT^®^, while the lowest MDC values in the CMJ was observed in WIMU^®^ (speed) and the video. The percentage of differences was less than 5% comparing CHRONOJUMP^®^ and the video in all types of jump. In all devices, the percentage of differences with CHRONOJUMP^®^ was lower as well as the more complex jump performed (CMJ and Abalakov), although the changes ranged between 24 and 34%.

Table 2 shows the inferential analysis between CHRONOJUMP^®^ and the rest of the devices according to each type of jump (SJ, CMJ, and ABK). The means comparison tests indicated statistically significant differences among instruments in all types of jumps (*p* = 0.001), with large differences (between 1.06 and 2.12) in almost all tests, except in the comparison between the video and CHRONOJUMP^®^ (trivial changes). Regarding reliability, the ICC showed high values in the simpler jumps (SJ and CMJ), except in the comparison with VERT^®^, in which the Abalakov jump recorded an ICC = 0.98. The video was the most reliable instrument in all types of jump (ICC = 0.98), followed by the WIMU^®^ using the flight time (ICC > 0.96). In the analysis of the WIMU^®^ device, the flight time was the more reliable option to measure whichever type of jump (ICC > 0.96), meanwhile the speed option was the worst (ICC < 0.84). Moreover, the more reliable ranges were found in the ICC over 0.90 due to the range not achieving values lower than 0.80.

**Figure 2:**
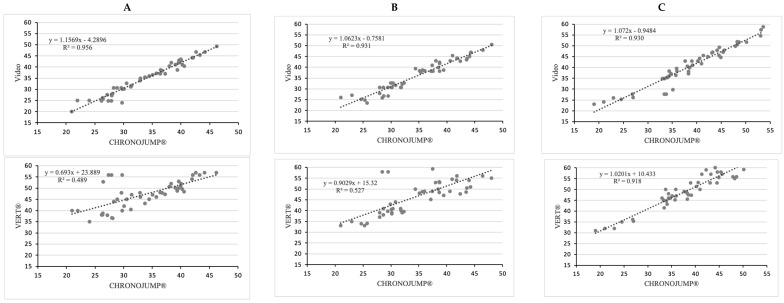
Relationship between CHRONOJUMP^®^ and other instruments (Video and VERT^®^) for (**A**) SJ: squat jump; (**B**) CMJ: countermovement jump; and (**C**) Abalakov jump. R^2^: Pearson’s multivariate coefficient of determination. The variables analyzed were the jump height measured in centimeters.

**Figure 3:**
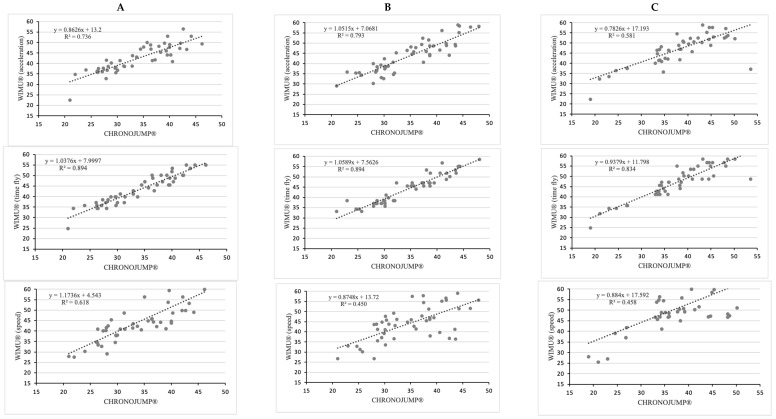
Relationship between CHRONOJUMP^®^ and WIMU^®^ in terms of acceleration, time fly or speed for (**A**) SJ: squat jump; (**B**) CMJ: countermovement jump; and (**C**) Abalakov jump. R^2^: Pearson’s multivariate coefficient of determination. The variables analyzed were the jump height measured in centimeters.

Concerning the level of agreement (R^2^), the higher values appeared with the use of video in all type of jumps (R^2^ > 0.93). The VERT^®^ device showed a higher level of agreement only for the Abalakov jump (R^2^ > 0.93) (Figure 2). The WIMU^®^ device recorded the higher agreement values when the flight time was set, especially in SJ and CMJ (R^2^ = 0.894) (Figure 3).

**Figure 4:**
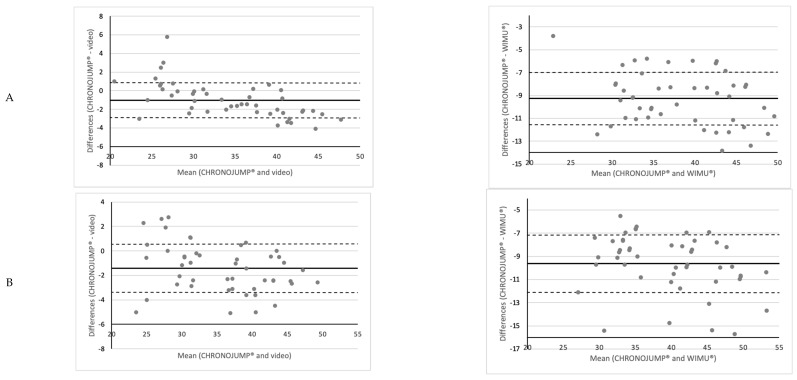

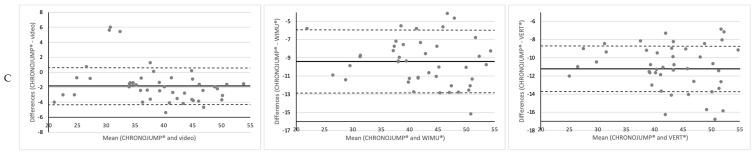
Bland–Altman plots comparing CHRONOJUMP^®^ with video and WIMU^®^ (time fly) according to time fly in (**A**) SJ: squat jump, and (**B**) CMJ: countermovement jump and also the VERT^®^ in (**C**) Abalakov jump. The variables analyzed were the jump height measured in centimeters.

Analyzing the bias among instruments, the Bland–Altman plot (Figure 4) showed a systematic error related to an overestimation in jump height in all devices compared to the CHRONOJUMP^®^. Compared to the video, the systematic error was less than two centimeters in the SJ and CMJ. However, the comparison with the WIMU^®^ (based on the flight time option) showed a difference of over nine centimeters for the SJ and CMJ. Finally, the error with VERT^®^ in the Abalakov jump was over 11 cm. This error remained constant regardless of the jump height.

## 4. Discussion

The aim of this study was to examine the concurrent validity and reliability of multiple devices to measure jump height in men’s handball players. Commensurate with a proper monitoring process, especially in jumps, it is necessary to analyze the outcomes from different devices in terms of an accurate and effective recording of training load. Overall, the instruments used had a lower minimum detectable change, especially in more simple executions (SJ and CMJ). Furthermore, there were statistically significant differences among devices, although higher concordance values were found between CHRONOJUMP^®^ with video and WIMU^®^ (flight time), especially in the SJ and CMJ. Moreover, the overestimation observed in all the devices remained constant independently of jump height.

Comparing with the CHRONOJUMP^®^ mat and based on the ICC values, the video appears to be the best device to measure jump height regardless of the type of jump. Following it, the WIMU^®^ device, specifically flight time, is considered a suitable tool, better than the acceleration or speed option. These differences may be related to the mathematical operations from the device. The indirect processes could be responsible for this variation [38,39]. Finally, although the VERT^®^ system also showed lower ICC values, this result changed in the Abalakov jump, perhaps influenced by the position of the device on the body, and the jump movement patterns [14,15]. Therefore, although the video recording system seems to be the most accurate and reliable option for the measurement of jump height, the calculation of said variable through the time of flight measured with the WIMU device can solidly fulfill this task, simplifying both the logistics and the environment in which this type of jumping experiment can be carried out.

Concerning the type of jump, better values of ICC were found in less complex jumps (SJ and CMJ), except in the comparison between CHRONOJUMP^®^ and VERT^®^ in the ABK jump. These results could be explained by the device location and the absence (SJ and CMJ) or implication (ABK) of the arms. In this respect, the location of the WIMU^®^ device in the interscapular area allows the recording of the jump due to the arm movement earlier than the VERT^®^, that locates the device on the hip [33], a joint away from trunk flexion and vertical acceleration [37]. It has been shown that the location of the device relative to the center of mass can alter the time-of-flight data and thus the jump height results, so the results appear to be consistent with this statement [40]. Based on the level of agreement (R^2^), the video also showed the best concordance in all types of jumps, and the WIMU^®^ device recorded the higher values when the flight time was set, especially in SJ and CMJ. As a consequence, for the calculation of the jump height, the execution of jumps with low technical complexity such as the SJ and the CMJ is recommended, reducing inter- and intra-subject variability for greater objectivity of the results obtained.

Regarding the graphical representations (Bland–Altman plots), a general and constant overestimation was found between CHRONOJUMP^®^ and the rest of the devices. In this respect, the video showed a minimum overestimation of less than two centimeters in all types of jumps. This difference may be due to the fact that the CHRONOJUMP^®^ contact platform has a very high sampling frequency (recording one datum every millisecond), while the video only records at 240 frames per second. In that vein, the WIMU^®^ showed another overestimation of slightly over nine centimeters in all jumps. The WIMU^®^ estimates vertical jump height using the time difference between the positive (propulsive phase of the jump) and negative (landing) peaks of vertical velocity. However, the maximal positive velocity is normally achieved shortly before the takeoff, and the maximal negative velocity shortly after landing, which inevitably causes the flight time recorded by WIMU^®^ to be longer than the effective flight time. Finally, the VERT^®^ device showed an overestimation of over 11 cm in ABK compared to CHRONOJUMP^®^. Therefore, both the sampling frequency and the data collection method of each of the devices must be previously known in order to be able to analyze the results obtained taking into account the error biases of each of them.

Despite these findings, some limitations and proposals for future research should be taken into account. Mainly, based on physical performance tests, these results (jump height) cannot be extrapolated to real game situations due to the absence of data concerning the horizontal vector, the common skills performed during the game (jumping with one leg), or the interactions with the opponent, etc. Therefore, future studies should be developed to show specific data in real conditions, both in training and competitions. On the other hand, previous studies have identified a jump height overestimation from the flight time calculation, so much caution should be taken into consideration to establish a performance profile related to the jump height. Finally, the concurrent validity and reliability of these devices in a sample of female or in power-based athletes are unknown. In addition, the position of the devices needs further investigation, especially in jumping actions.

## 5. Conclusions and Practical Applications

Coaches and trainers should be cautious when selecting the measuring instrument to assess and monitor athletes’ jump performance. The risk of collecting confusing data leads to misinterpretations that can affect the quality of training, and therefore the athletes’ health and performance. In this study, the usefulness of some devices was evaluated, also in terms of jump height measures. The jumping test used, the coaching environment, and the performance goals can influence the device selection. Moreover, the differences in jump height are perfectly calculated, so any coach can use the most appropriate device and calculate the real jump height derived by knowing the differences among instruments.

## Figures and Tables

**Table 1:** Jump height calculated by mean ± standard error of the mean and (minimum detectable change).

	CHRONOJUMP^®^	WIMU^®^ Acceleration	WIMU^®^ Time Fly	WIMU^®^ Speed	Video	VERT^®^
SJ	33.77 ± 0.93 (2.59)	42.33 ± 0.94 (2.61)	43.04 ± 1.03 (2.84)	44.17 ± 1.40 (3.87)	34.77 ± 1.11 (3.07)	47.29 ± 0.93 (2.57)
CMJ	34.84 ± 0.96 (2.67)	43.70 ± 1.14 (3.16)	44.45 ± 1.08 (2.99)	44.19 ± 1.26 (3.48)	36.25 ± 1.06 (2.94)	46.77 ± 1.20 (3.32)
Abalakov	38.64 ± 1.18 (3.28)	47.44 ± 1.21 (3.36)	48.04 ± 1.21 (3.36)	51.75 ± 1.54 (4.27)	40.48 ± 1.31 (3.63)	49.85 ± 1.26 (3.48)

Notes: Abalakov: Abalakov jump; CMJ; countermovement jump; MDC: minimum detectable change; SJ: squat jump.

**Table 2:** Inferential data (*p* value, d Cohen, ICC and R^2^) after the comparison between Chronojump^®^ and the rest of devices.

CHRONOJUMP^®^ and WIMU^®^ Acceleration	*p* Value	*d* Cohen	ICC (95% CI)	R^2^
SJ	0.001	−1.33	0.92 (0.86–0.96)	0.736
CMJ	0.001	−1.12	0.94 (0.86–0.96)	0.793
Abalakov	0.001	−1.06	0.87 (0.76–0.92)	0.581
CHRONOJUMP^®^ and WIMU^®^ Time fly				
SJ	0.001	−1.38	0.97 (0.95–0.98)	0.894
CMJ	0.001	−1.36	0.97 (0.94–0.98)	0.894
Abalakov	0.001	−1.14	0.96 (0.92–0.98)	0.834
CHRONOJUMP^®^ and WIMU^®^ Speed				
SJ	0.001	−1.28	0.84 (0.72–0.91)	0.618
CMJ	0.001	−1.20	0.79 (0.62–0.88)	0.450
Abalakov	0.001	−1.38	0.79 (0.63–0.88)	0.458
CHRONOJUMP^®^ and Video				
SJ	0.001	−0.14	0.98 (0.97–0.99)	0.956
CMJ	0.001	−0.20	0.98 (0.96–0.99)	0.931
Abalakov	0.001	−0.20	0.98 (0.96–0.99)	0.930
CHRONOJUMP^®^ and VERT^®^				
SJ	0.001	−2.12	0.82 (0.68–0.90)	0.489
CMJ	0.001	−1.58	0.83 (0.70–0.91)	0.527
Abalakov	0.001	−1.33	0.98 (0.96–0.99)	0.918

Notes: Abalakov: Abalakov jump; CMJ; countermovement jump; ICC: intraclass correlation coefficient; MDC: minimum detectable change; R^2^: coefficient of determination; SJ: squat jump.

## Data Availability

The datasets generated from the study are available from the corresponding author on reasonable request.
